# The Application of Dentistry to the Detection of Crime

**Published:** 1885-05

**Authors:** 


### The Application of Dentistry to the Detection of Crime.

Mr. Robert Reid (*Journal of British Dental Association*, Sept. 1884,) enlarges upon this subject, and publishes an instructive case of murder. The room where the murdered man had been struck down was strewn with detached portions of the upper jaw bone, the largest of which extended from the second bicuspid on the left side to the canine tooth on the right, inclusive. The alveoli of the right and left upper laterals were occupied by left central and lateral incisors of the lower jaw. Among the detached teeth were right and left upper lateral incisors, corresponding exactly in shape and size with their fellows. On examination of the accused person, his central and lateral incisors of the left side of the lower jaw were found to have been recently removed, and the corresponding teeth removed from the jaw of the deceased were found to fit into the proper alveolar cavities of the accused.—*Journal of American Dental Association.*

